# Unitary transformation for Poincaré beams on different parts of Poincaré sphere

**DOI:** 10.1038/s41598-020-71189-2

**Published:** 2020-08-28

**Authors:** Xibo Sun, Yuanchao Geng, Qihua Zhu, Wanqing Huang, Ying Zhang, Wenyi Wang, Lanqin Liu

**Affiliations:** Research Center of Laser Fusion, China Academy of Engeering Physics, Mianyang, 621900 China

**Keywords:** Optical manipulation and tweezers, Transformation optics, Optical techniques

## Abstract

We construct an experimental setup, consisting of conical refraction transformation in two biaxial cascade crystals and 4f-system, to realize Unitary transformation of light beam and the manipulation of Poincaré beams on the different parts of Poincaré sphere. The spatial structure of the polarization can be controlled by changing the polarization of the incident beam or rotating the angle between these two crystals. The beams with different SoPs covering the full-Poincaré sphere, part-Poincaré sphere and one point on the sphere are generated for the different angles between crystals. The Unitary transformation of light beam is proposed in the experiment with the invariant intensity distribution. Subsequently, the spin angular momentum is derived from the distribution of polarization measured in our experiment. Moreover, the conversion between orbital angular momentum and spin angular momentum of light beam is obtained by changing the angle between crystals. And the conversion progress can also be influenced by the polarization of incident beam. We realized the continuous control of the spatial structure of the angular momentum density, which has potential in the manipulation of optical trapping systems and polarization-multiplexed free-space optical communication.

## Introduction

Up to now, as an intrinsic and fundamental vectorial nature of light, the SoP has attracted wide attention owing to its important role in propagation behaviors, focusing properties, and light-matter interactions. In 2010, Beckely introduced and demonstrated one kind of novel vector beams, named full Poincaré (FP) beams, in the experiment^1,2^. One dominant characteristic of FP beams is that the states of polarization (SoPs) in the cross-section cover the entire surface of Poincaré sphere. As a result, any SoP on Poincaré sphere can be found in the FP beams

Due to the special SoP distribution, The FP beams have potential for many practical applications in beam shaping^3,4^, manipulation of particles^5^, generating transverse spin angular momentum^6–8^ and significantly reducing turbulence-induced scintillation^9,10^. High-order FP beams have been proposed to have benefit in achieving a smooth flat-top transverse profile with steep edge roll-off^11,12^. The orders herein are determined by the number of times that the SoP covers all possible polarization states over a Poincaré sphere.

Accordingly, there are numerous methods and devices proposed in previous researches for generating the FP beams to achieve further applications, including spatial light modulators^13–15^, uniaxial crystals^16,17^, q-plates and dielectric metasurface^18–20^, geometric phase control inside a laser cavity^21^, and so forth.

Conical refraction was demonstrated to be a method to manipulate the SoP of beams as an intrinsic property of biaxial crystals. The cascade conical diffraction phenomenon was comprehensively studied in detail elsewhere^22–25^. The further manipulation of SoP were realized, with the multiple-concentric-rings intensity distribution. Based on the characters of the conical refraction, in our previous research in 2017^26^, one kind of structured light beam with periodical polarization and phase singularities was theoretically and experimentally obtained from a setup consisting of conical refraction transformation and 4f-system. This beam was verified to be one kind of full Poincaré beams in the research^27^, and the effects of using cascade biaxial crystals on the manipulation of polarization was investigated. The Jones matrix for this transformation was presented to be a Unitary matrix independent of the angle of two crystals, resulting in the manipulation of SoP with the invariant intensity of near-field. The beams with different SoPs covering the full-Poincaré sphere, part-Poincaré sphere and one point on the sphere were generated for the different angles between crystals.

For further research, we conduct an experimental study of the manipulation of polarization in this work by the method mentioned above. The measurement of the SoP were performed to represent the effect of the Unitary transformation with different SoP of the input beam and the angle between two crystals. Then, the Stokes parameters were calculated and projected on Poincaré sphere to visualize the distribution of the SoP. Since the spin angular momentum has linear relationship with the SoP^6,7^, we subsequently concentrate on the local and global angular momentums of output beam to evaluate the potential application prospect of the proposed setup.

## Theoretical analysis

**Figure 1:**
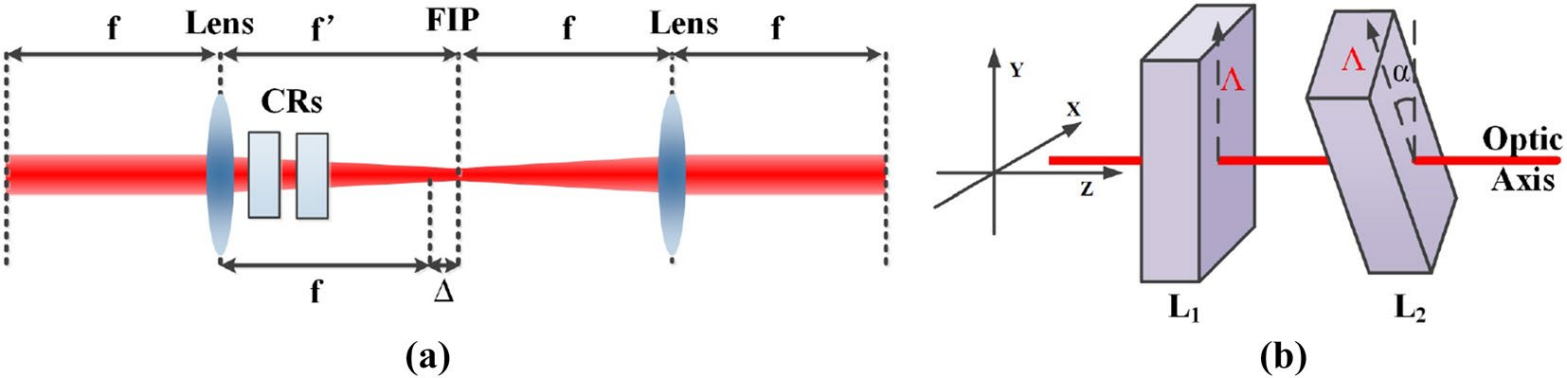
Schematic of (**a**) the optical design to realize the modulation of SoP and (**b**) the cascade crystals with angle between pseudo-vectors $\Lambda$, resulting in the lateral shift. *FIP* focal image plane, *CRs* Conical Refraction crystals. Ploted by Microsoft Office Visio 2010.

In this research, the common focus of a 4f system was set on the focal image plane (FIP) of the two cascade biaxial crystals with the length of $L_1$ and $L_2$, as shown in Fig. 1, resulting in the Unitary transformation of light field. The optic axis of the two biaxial crystals, which the crystal are rotated around, are parallel with each other and the principal refractive indices $n_1<n_2<n_3$.

According to the previous studies by Berry and Phelan^25,28^, the Fourier transform ${\mathbf{a}}({\mathbf{P}},z)$ of the electric field ${{\mathbf{E}}_{{\mathbf{CR}}}}({\mathbf{r}},Z)$, which is transformed by conical diffraction at the plane with the length of *Z* from FIP can be expressed as^27^

1 $$\begin{aligned} {\mathbf{a}}({\mathbf{P}},z) = \exp \left( { - \frac{1}{2}ik{P^2}Z} \right) \cdot {\mathbf{T}} \cdot {\mathbf{a}}({\mathbf{P}},0) \end{aligned}$$

${\mathbf{P}}$ is the dimensionless transverse wavevector of plane waves ${\mathbf{k}}$. ${\mathbf{a}}({\mathbf{P}},0)$ is the Fourier transform of the input electric field ${{\mathbf{\mathrm{E}}}_{in}}$. *k* is the wavenumber in crystal satisfying $k = {n_2}{k_0}$.

The transformation matrix **T** represents the manipulation of the beam passing through cascade biaxial crystals.

2 $$\begin{aligned} {\mathbf{T}} = {\mathbf{R}}(\alpha ){\mathbf{C}}(P,\phi - \alpha ,{R_{02}}){\mathbf{R}}( - \alpha ){\mathbf{C}}(P,\phi ,{R_{01}}) \end{aligned}$$

where, ${\mathbf{R}}\left( \alpha \right)$is the rotation matrix satisfying $${\mathbf{R}}\left( \alpha \right) = \left[ {\begin{array}{*{20}{c}} {\cos \alpha }&{}{ - \sin \alpha }\\ {\sin \alpha }&{}{\cos \alpha } \end{array}} \right]$$. The matrix ${\mathbf{C}}\left( {P,\phi ,{R_0}} \right) = \cos \left( {kP{R_0}} \right) {\mathbf{I}} - i\sin \left( {kP{R_0}} \right) {\mathbf{M}}\left( \phi \right)$ is the transformation matrix for single biaxial crystal with the length of *L*. ${\mathbf{r}} = \left( {r,\phi } \right)$ is the position at the transverse plane in cylinder coordinates. The matrix ${\mathbf{M}}\left( \phi \right)$ equals $$\left[ {\begin{array}{*{20}{c}} {\cos \phi }&{}{\sin \phi }\\ {\sin \phi }&{}{ - \cos \phi } \end{array}} \right]$$ and ${\mathbf{I}}$ is the identity matrix by $2 \times 2$. ${R_0} = \left( \sqrt{\left( {{n_3} - {n_2}} \right) \left( {{n_2} - {n_1}} \right) } /{n_2}\right) L$ is the radius of the refracted ring beam beyond the crystal.

Through the second lens, the light field in the output plane of our system can be expressed as^27^,

3 $$\begin{aligned} {{\mathbf {\mathrm{E}}}_{out}}({\mathbf {r}}) = {\mathbf {T}} \cdot {{\mathbf {\mathrm{E}}}_{in}}({\mathbf {r}}) = {E_{in}}{\mathbf {T}} \cdot \left[ {\begin{array}{*{20}{c}} {{e_x}} \\ {{e_y}} \end{array}} \right] \end{aligned}$$

${\left[ {{{e_x}}\quad {{e_y}}} \right] ^T}$ represents the SoP of input beam, for instance, ${\left[ {{{1}}\quad {{i}}} \right] ^T}$ and ${\left[ {{{0}}\quad {{1}}} \right] ^T}$ denote the left-handed circular and y-linear polarization, respectively.

The transformation matrix ${\mathbf {T}}$ has been demonstrated to be a Unitary matrix satisfying ${{\mathbf {T}}^*} \cdot {\mathbf {T}} = {\mathbf {\mathrm{I}}}$ with arbitrary rotation angle $\alpha$, as shown in Fig. 1b.

Especially for the case that the cascade crystals have equal length as well as the equal $R_0$. The elements of matrix *T* expressed as $T_{11}$, $T_{12}$, $T_{21}$ and $T_{22}$ can be derived as

4 $$\begin{aligned} \begin{array}{l} {T_{11}} = [(1 + \cos (2kP{R_0})) - \cos (\alpha )(1 - cos(2kP{R_0})) - i(\cos (\phi ) + \cos (\alpha + \phi ))\sin (2kP{R_0})]/2\\ {T_{12}} = [\sin (\alpha )(1 - \cos (2kP{R_0})) - i(\sin (\phi ) + \sin (\alpha + \phi ))\sin (2kP{R_0})]/2\\ {T_{21}} = [ - \sin (\alpha )(1 - \cos (2kP{R_0})) - i(\sin (\phi ) + \sin (\alpha + \phi ))\sin (2kP{R_0})]/2\\ {T_{22}} = [(1\mathrm{{ + }}\cos (2kP{R_0})) - \cos (\alpha )(1 - \cos (2kP{R_0})) + i(\cos (\phi ) + \cos (\alpha + \phi ))\sin (2kP{R_0})]/2 \end{array} \end{aligned}$$

Therefore, the Stokes parameters ${S_0},{S_1},{S_2},{S_3}$ could be calculated from these intensity distributions by the formula as follows.

5 $$\begin{aligned} \begin{aligned} {S_0} =\,&{\left| {{E_x}} \right| ^2} + {\left| {{E_y}} \right| ^2}{\text { = }}{I_{0^\circ }} + {I_{90^\circ }} \\ {S_1} =\,&{\left| {{E_x}} \right| ^2} - {\left| {{E_y}} \right| ^2} = {I_{0^\circ }} - {I_{90^\circ }} \\ {S_2} =\,&2{\text {Re}} \left[ {{E_x}^*{E_y}} \right] = {I_{45^\circ }} - {I_{ - 45^\circ }} \\ {S_3} =\,&2{\text {Im}} \left[ {{E_x}^*{E_y}} \right] = {I_R} - {I_L} \\ \end{aligned} \end{aligned}$$

When the incident beam is in right-handed circular polarization, the Stokes parameters representing the polarization of the beam can be subsequently obtained as follows

6 $$\begin{aligned} \begin{array}{l} {S_0} = {I_{\mathrm{{in}}}}\\ {S_1} = {I_{\mathrm{{in}}}}\sin (2kP{R_0})[ - \sin (2\alpha + \phi ) - \sin (\alpha + \phi ) + (\sin (\alpha + \phi ) + \sin (\phi )/2 + \sin (2\alpha + \phi )/2)(1 + \cos (2kP{R_0}))]\\ {S_2} = {I_{\mathrm{{in}}}}\sin (2kP{R_0})[\cos (2\alpha + \phi ) + \cos (\alpha + \phi ) - (\cos (\alpha - \phi ) + \cos (\phi )/2 + \cos (2\alpha + \phi )/2)(1 + \cos (2kP{R_0}))]\\ {S_3} = {I_{\mathrm{{in}}}}[1 - \sin {(2kP{R_0})^2}(1 + cos(\alpha ))] \end{array} \end{aligned}$$

**Figure 2:**
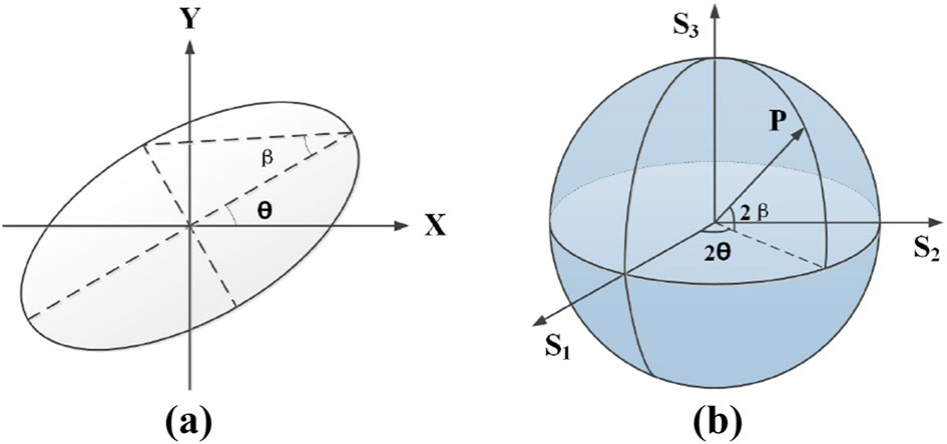
The representation of the polarization states. (**a**) the polarization ellipse and (**b**) the Poincaré sphere. Ploted by Microsoft Office Visio 2010.

As a result, the azimuth angle $\theta$ and the ellipticity angle $\beta$ of the polarization ellipse, which is also a useful way to visualize polarization, can be confirmed uniquely by means of measuring the Stokes parameters, as shown in Fig. 2.

7 $$\begin{aligned} \begin{array}{l} 2\theta = \arctan (\frac{{{S_2}}}{{{S_1}}}) =\arctan \left[ \frac{{ - \cos (\alpha + \phi ) - \cos (2\alpha + \phi ) + 2\cos (\alpha + \phi )(1 + \cos (\alpha )) \cos {{(kP{R_0})}^2}}}{{ - \cos (\alpha + \phi ) - \cos (2\alpha + \phi ) + 2\sin (\alpha + \phi )(1 + \cos (\alpha ))\cos {{(kP{R_0})}^2}}}\right] \\ 2\beta = \arcsin \left( \frac{{{S_3}}}{\sqrt{{S_1}^2+{S_2}^2}}\right) =\arcsin (1 - \sin {(2kP{R_0})^2}(1 + \cos (\alpha ))) \end{array} \end{aligned}$$

In which, the range of ellipticity angle $\beta \in [\alpha /2 - \pi /4,\pi /4]$ reveals the relation of the proportion of SoPs projected on the Poincaré sphere and the rotation angle $\alpha$ of second crystal. The effects are similar for the cases that the incident beam is in different polarization.

## Experimental setup

**Figure 3:**
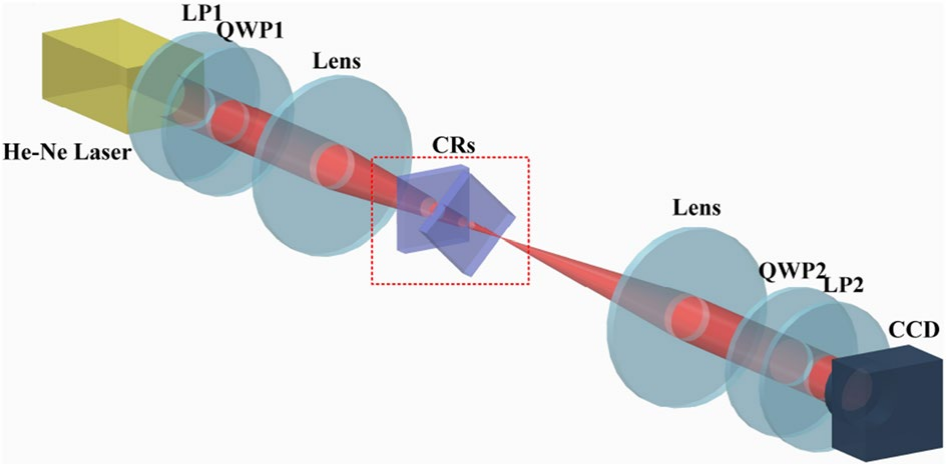
Schematic overview of the experimental setup. *LP* Linear Polarizer, *QWP* Quarter-Wave Plate, *CRs* Conical Refraction crystals. Created by Creo 2.0.

A collimated random polarized He-Ne laser at the wavelength of 632.8nm was used as the light source, and the beam waist radius was 1mm at the input plane. As shown in Fig. 3, a linear polarizer (LP1) and a quarter-wave plate (QWP1) were adopted to control the SoP of the input beam. The 4f system consisted of a pair of confocal lenses with the focal length of 200 mm. The experiments were performed with two 5 mm long $KGd(WO _4) _2$ crystals as the biaxial crystals . The optic axes of these cascade crystals were parallel to the transmission direction of the light beam, and the second crystals could be rotated through angle $\alpha$ around the axis. Subsequently, the output beam was projected onto the CCD camera at the back focal plane. The quarter-wave-plate (QWP2) and the linear polarizer (LP2) positioned before CCD were applied as SoP detectors to measure the intensity distribution of both linearly and circularly polarized components, denoted by ${I_\varphi }$ (the angle $\varphi$ between polarizing axis and x-axis satisfying $\varphi = 0^\circ ,45^\circ ,90^\circ , - 45^\circ$), $I_R$ (in right-hand) and $I_L$ (in left-hand).

## Results and discussions

### Right-handed circularly polarized input beam

Firstly, we set the axis of P1 parallel with the x-axis and the fast axis of QWP at $45^\circ$ direction to generate the right-handed circularly polarized input beam. Then the intensity distributions mentioned above were recorded after rotating second crystals around the optic axis in a series of directions $\alpha = 0,\pi /3,\pi /2,2\pi /3,5\pi /6,\pi$.

**Figure 4:**
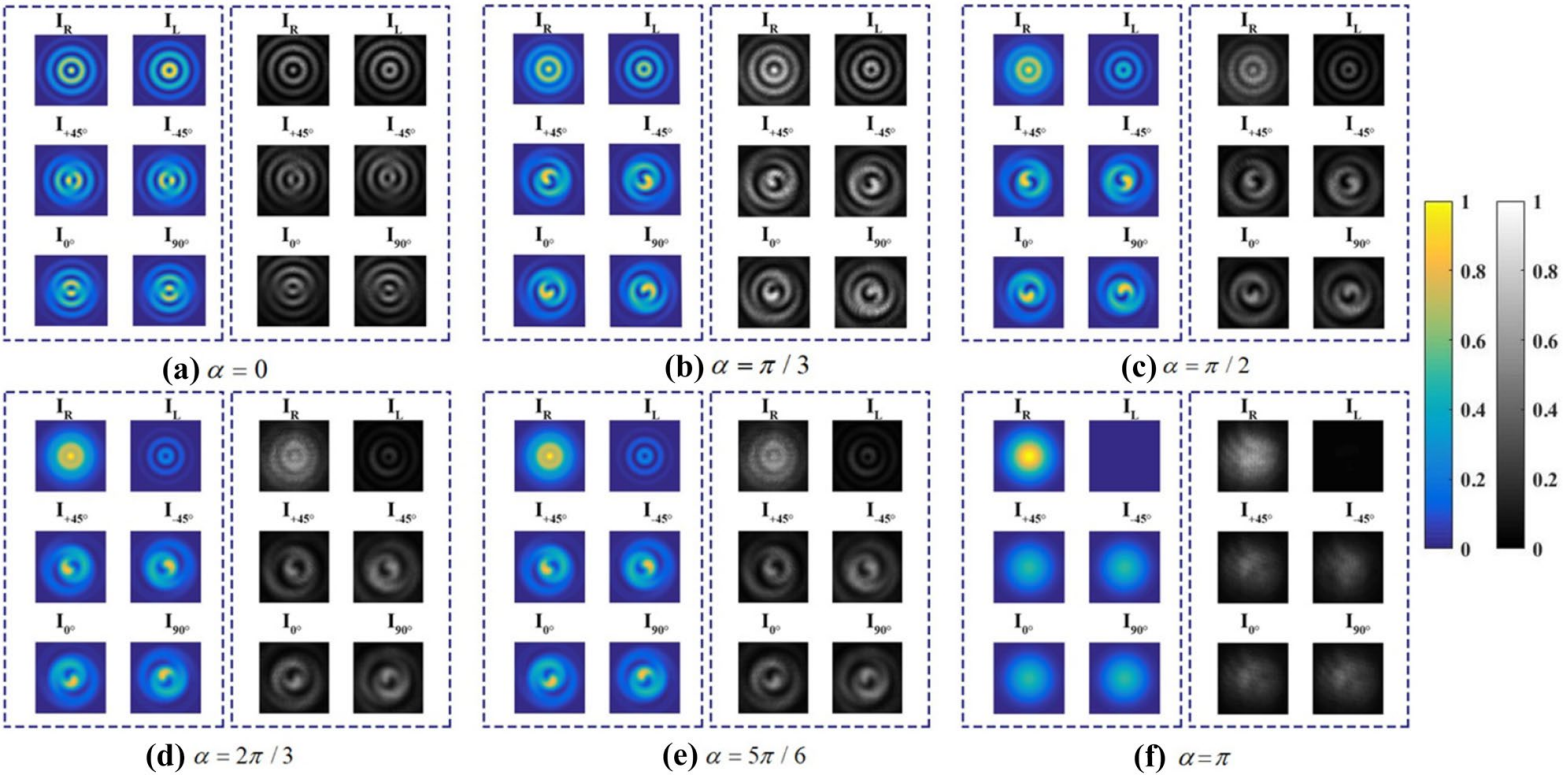
The intensity distributions in different polarization states after a polarization detector for a series of crystal directions (**a**–**f**) $\alpha = 0,\pi /3,\pi /2,2\pi /3,5\pi /6,\pi$ . The left column in every subfigure presents the theoretical result, while the right column shows the experimental result. Created by Matlab R2016a (9.0.0.341360),similarly hereinafter.

**Figure 5:**
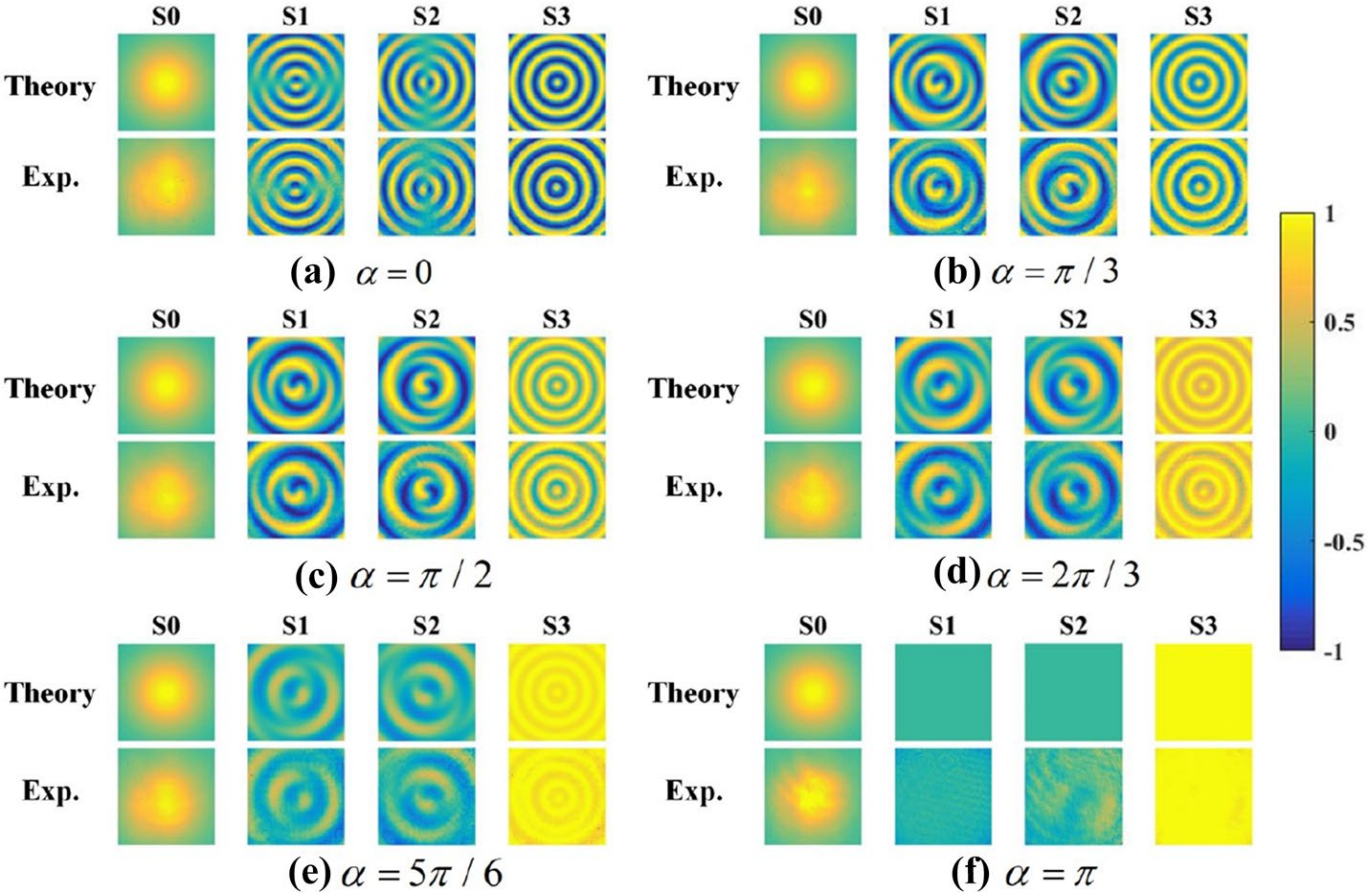
The Stokes parameters of output beams for a series of crystal directions (**a**–**f**) $\alpha = 0,\pi /3,\pi /2,2\pi /3,5\pi /6,\pi$ . The top row is the theoretical results, and the bottom row is the corresponding experimental results.

The intensity profile distributions obtained from experiments and theoretical analysis are shown in Fig. 4. The Stokes parameters of the output beam could be calculated from these intensity distributions by Eq. (5). And, Fig. 5 illustrates the numerical and experimental transverse patterns of Stokes parameters.

**Figure 6:**
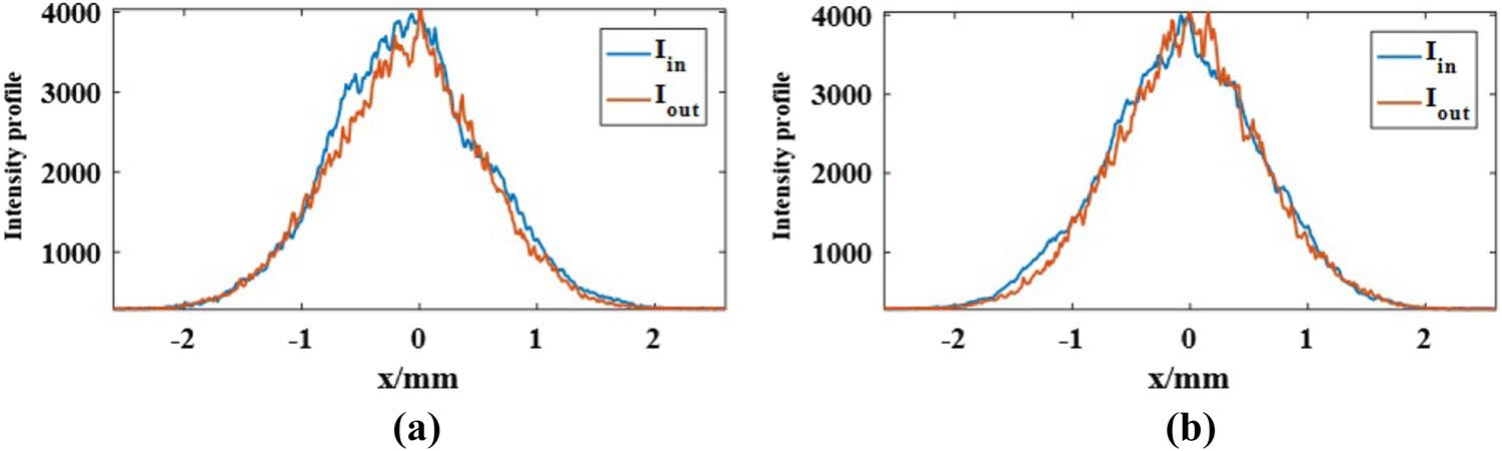
The intensity profiles of the incident beam and the output beams along (**a**) the x-axis and (**b**) the y-axis.

It could be found that the intensity distribution represented by the ${S_0}$ remains in Gaussian shape for different angle, while the SoP has already been manipulated with the inhomogeneous and variant distribution. Figure 6 compared the intensity distribution of the incident beam and the output beams for the case $\alpha = 0$ to demonstrate that the manipulation of the light could satisfy the Unitary transformation.

**Figure 7:**
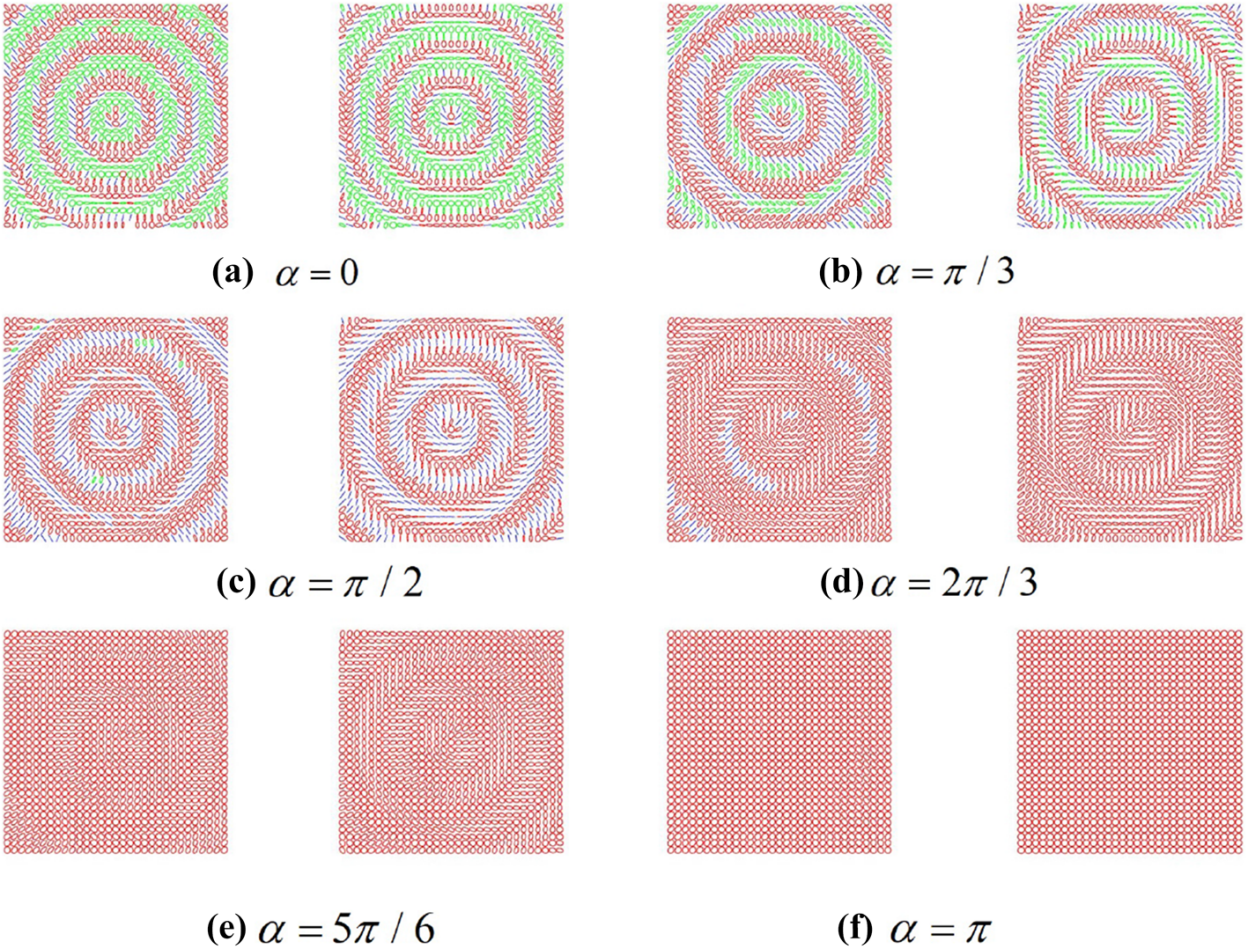
The polarization ellipses distribution of the output beams for different angle of crystal (**a**–**f**) $\alpha = 0,\pi /3,\pi /2,2\pi /3,5\pi /6,\pi .$

According to the Stokes parameters analysis, the polarization ellipses of the output beam are plotted in the Fig. 7 to visualize the change of the SoPs with the angle $\alpha$. The right and green ellipses represent the right-handed polarization and the left-handed polarization, respectively. While, the blue lines represent the linear polarization.

**Figure 8:**
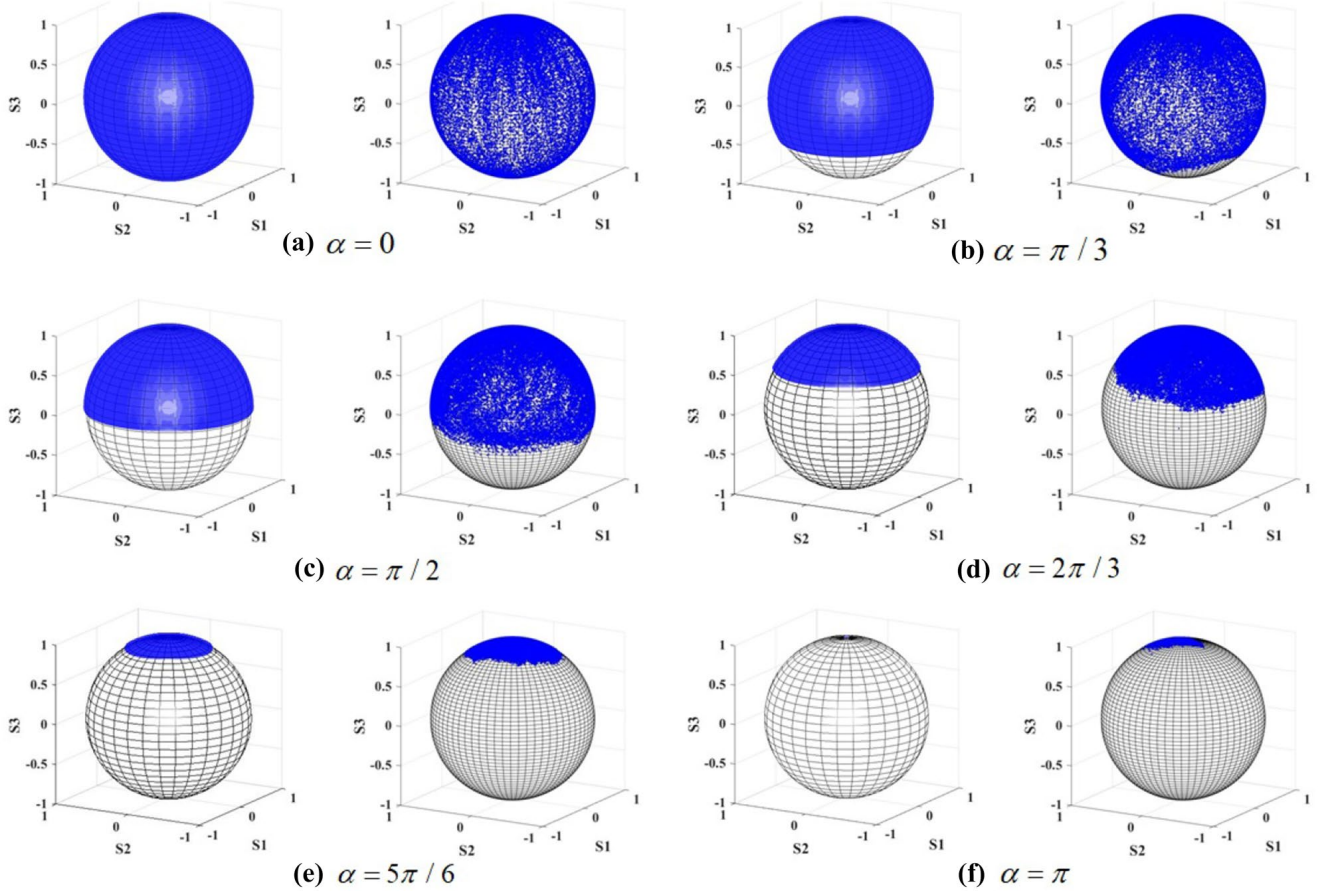
The distribution of SoP on Poincaré sphere for different angle of crystal (**a**–**f**) $\alpha = 0,\pi /3,\pi /2,2\pi /3,5\pi /6,\pi$. The left column records the theoretical result, while the right column records the experimental result.

To visualize the SoP of the output beams, the Stokes parameters were projected on Poincaré sphere as shown in Fig. 8. It could be obviously found that the proportion covered by the SoP onto Poincaré sphere, would decrease significantly with the increase of angle $\alpha$ ($\alpha \le \pi$). Specially, when the pseudo-vectors of two crystals were parallel with each other in same direction($\alpha =0$), the SoP covered the whole Poincaré sphere resulting in a full-Poincaré beam. In contrast, when in opposite direction, the SoP would cover one point on Poincaré sphere resulting in homogeneous polarization. Meanwhile, when the second crystal is rotated through $\pi /2$ , the SoP would cover half of the sphere . According to the defined order of Poincaré beam, it might be related to fractional order Poincaré beams.

Figure 8 indicates some slight difference between the observed and the calculated results. The proportion on Poincaré sphere measured in the experiment is found to be larger than the calculated value. In our previous research, the proportion has been proposed to have certain relation with the angle $\alpha$. More specifically, the cone angle corresponding to the proportion equals $2(\pi - \alpha )$, and the symmetry axis of the proportion has been demonstrated in relation with the SoP of the input beam. According to the published conclusion, the rotation error coming from crystal carrier and the polarization error coming from P1 and QWP1 would result in the measurement error. In addition, some modulation of intensity distribution produced from the speck on the surface of elements and the window of CCD would also result in the additional error of SoP. The background noise and the modulation of the output beams would result in the additional statistical errors of the stokes parameters measured in the experiments. Consequently, the corresponding proportion projected on the Poincare sphere would increase in the same manner as image data obtained from Fig. 8. Moreover, the cut angle error of the crystal would cause the tilt of crystal and finally affect the actual length of the crystal in transmission route.

### Linearly polarized input beam

**Figure 9:**
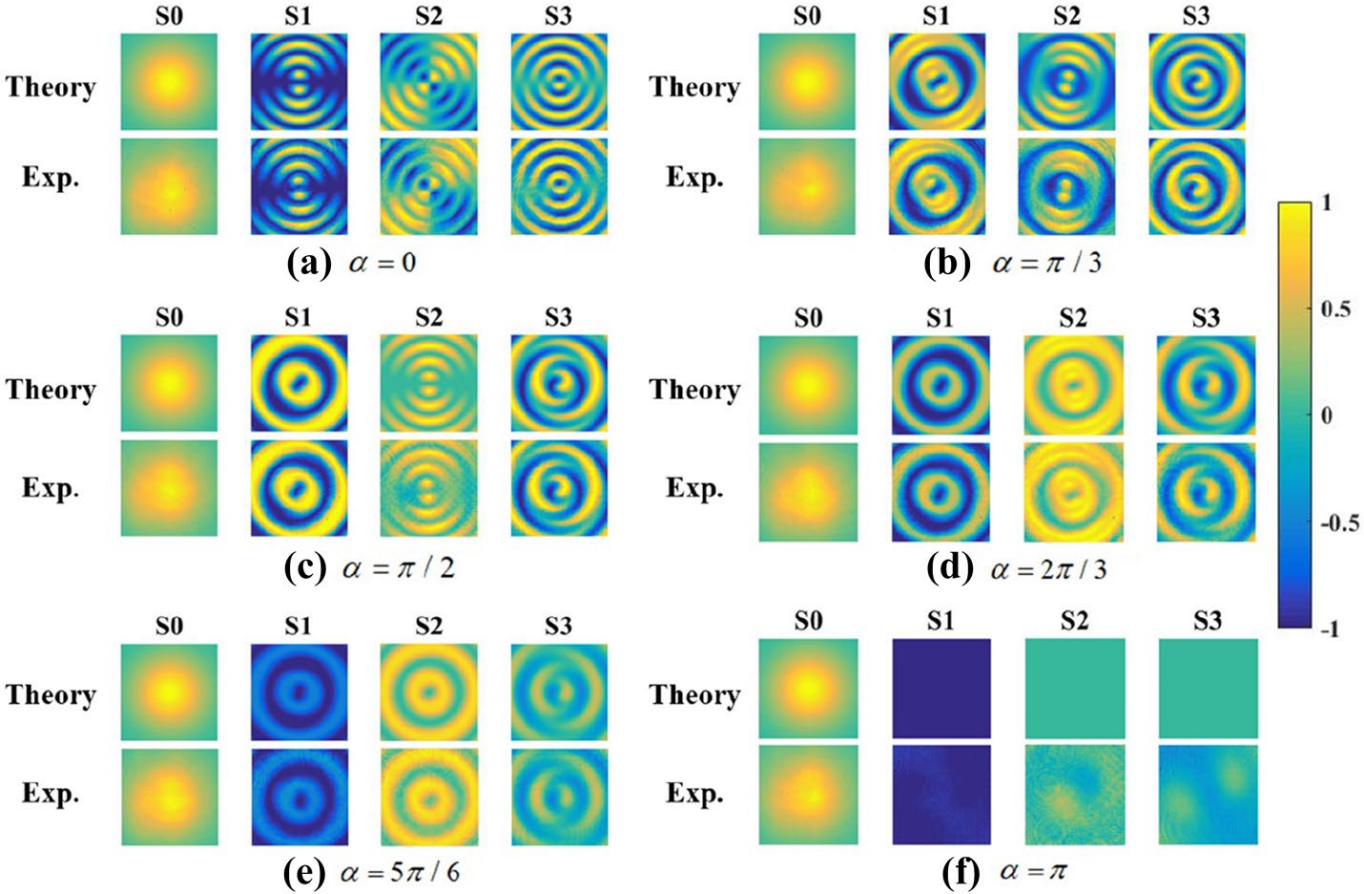
The Stokes parameters of output beams for a series of crystal directions (**a**–**f**) $\alpha = 0,\pi /3,\pi /2,2\pi /3,5\pi /6,\pi$ . The top row is the theoretical results, and the bottom row is the corresponding experimental results.

Then, we set the axis of P1 and the fast axis of QWP both parallel with x-axis. The intensity distributions mentioned above are recorded by the same method in the section 2. Figure 9 shows the numerical and experimental transverse pattern of Stokes parameters of the outputs. Similarly, the intensity distribution remains invariant for different angle, while the SoP has been changed. In addition, the inhomogeneous distribution are different from the case for circularly polarized incident beam. Obviously, the intensity distribution are independent with the angle $\alpha$ and SoP of incident beam, resulted from the Unitary transformation.

**Figure 10:**
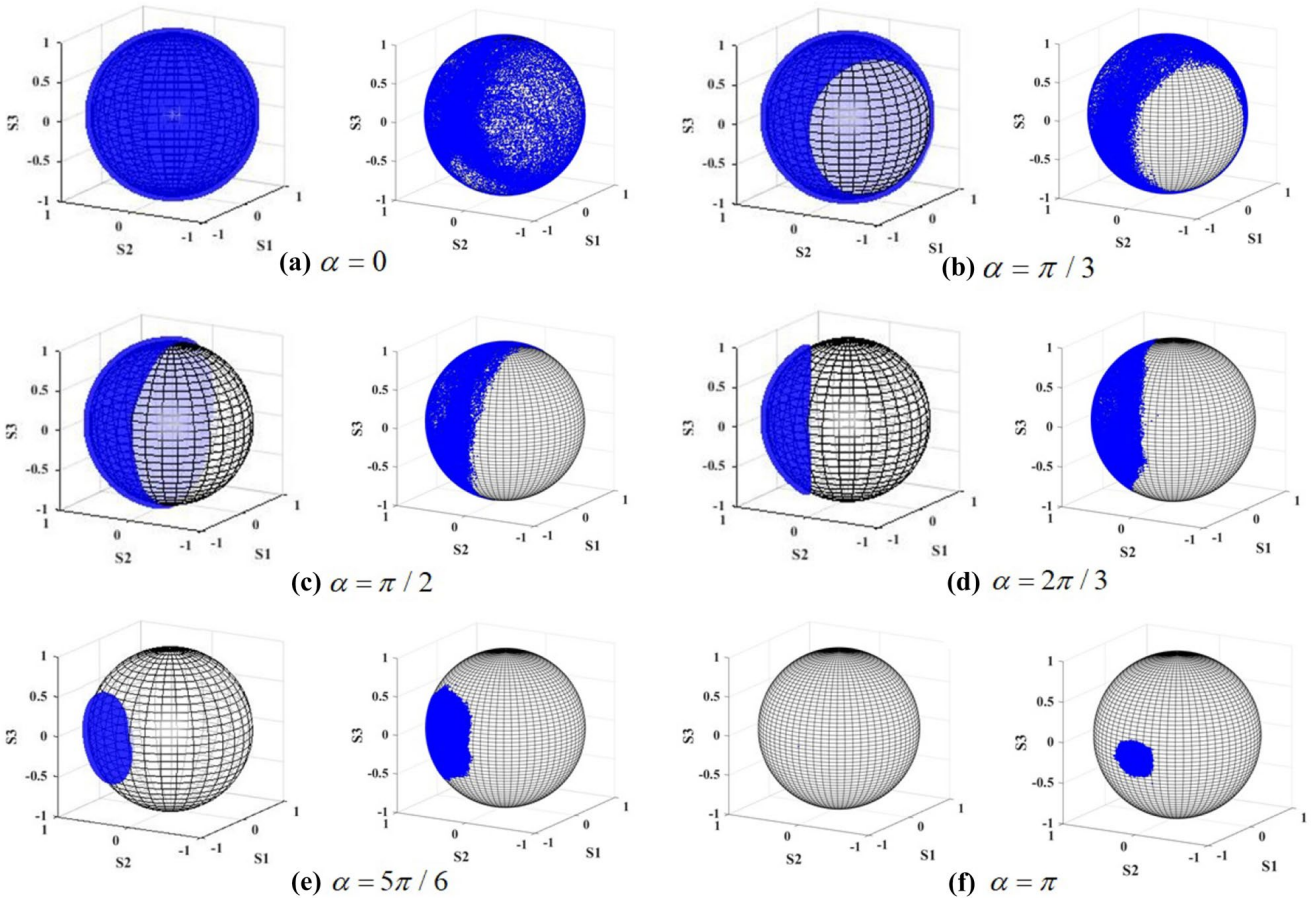
The distribution of SoP on Poincaré sphere for different angle of crystal (**a**–**f**) $\alpha = 0,\pi /3,\pi /2,2\pi /3,5\pi /6,\pi$ . The left column records the theoretical result, while the right column records the experimental result.

The projection of SoP on is presented in Fig. 10. Similarly with the discussion in last subsection, from Fig. 10 it can be obviously found that the proportion decreases as the angle $\alpha$ ($\alpha \le \pi$) increases. The SoP on the sphere covers the proportion with cone angle equaling $2(\pi - \alpha )$. Specially, when the second crystal is rotated through $0,\pi /2$ and $\pi$ , the SoP would respectively cover the whole, half and one point of the sphere.

The difference between the theoretical result and the experimental result remains for the same reasons that we analyze above.

## The extended research for the angular momentum

In the section above, we have demonstrated the light field distribution in the theoretical analysis of Ref.^27^. According to the light field distribution, the angular momentum (AM) distribution of the light field can be deduced, which has influence on the interaction between the light and the particle.

The AM of light can be separated into the orbital angular momentum (OAM) and the spin angular momentum (SAM)^29,30^. The separation of AM into spin and orbital parts is straightforward in paraxial monochromatic beams. The spin and orbital AM of light are separately observable properties in optics, which are associated with the polarization and the spatial distribution of the fields, respectively.

The local angular momentum densities have been intensively demonstrated in the context of both paraxial and nonparaxial beams, which can be expressed explicitly in the following form^31^:

8 $$\begin{aligned} OAM= & {} \frac{{{\varepsilon _0}}}{{2\omega }}{\mathop {\mathrm{Im}}\nolimits } (E_x^*{\partial _\varphi }{E_x} + E_y^*{\partial _\varphi }{E_y}) \end{aligned}$$

9 $$\begin{aligned} SAM= & {} \frac{{{\varepsilon _0}}}{\omega }{\mathop {\mathrm{Im}}\nolimits } (E_x^*{E_y}) \end{aligned}$$

**Figure 11:**
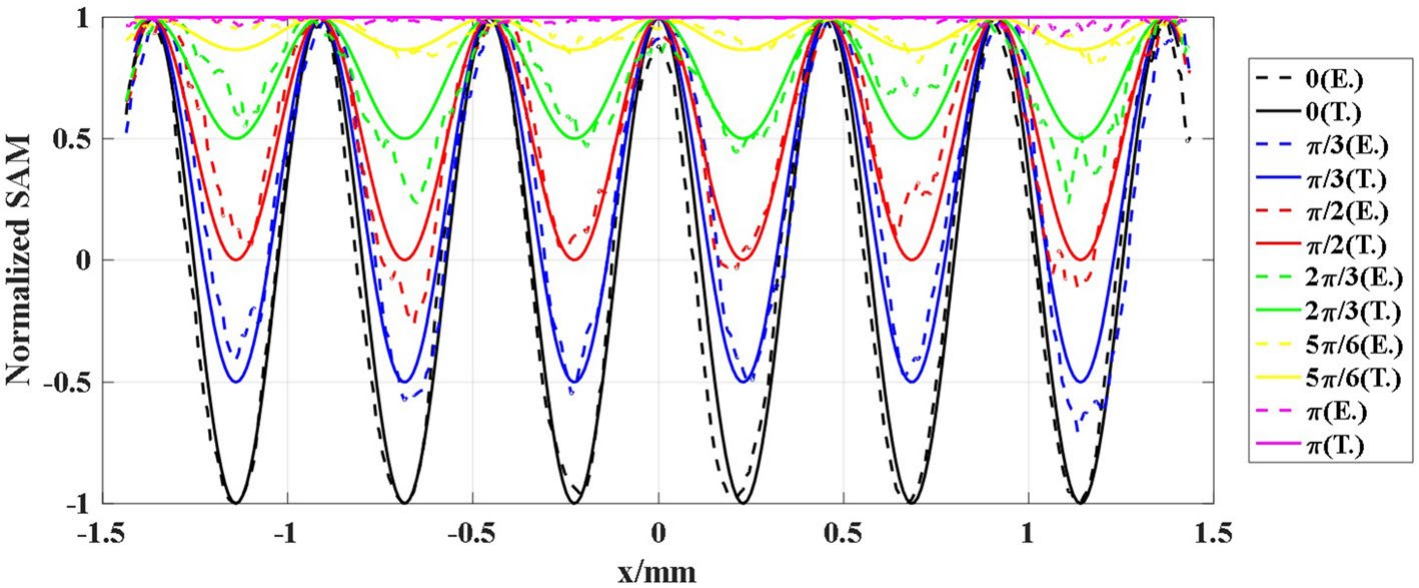
The profile of the SAM distribution along the radium direction. The solid lines are the theoretical results, and the dash lines are the corresponding experimental results.

According to the equations mentioned above, the SAM is proportional to the parameter ${S_3}$. As a result, from the Fig. 11 , we can find that SAM has the special periodicity along with the radial direction. As the angle between the cascade crystals is changed, the spatial periodicity remains invariant, while the range of the SAM is changed.

**Figure 12:**
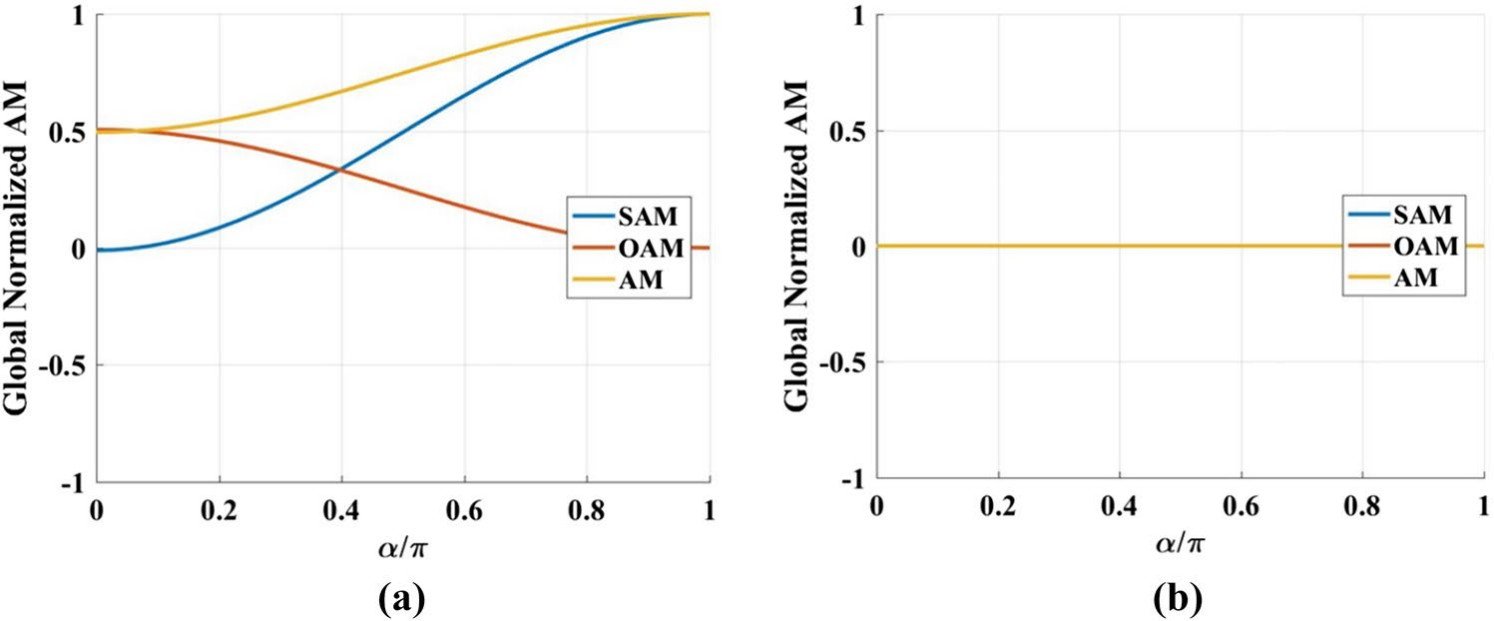
Global AM for (**a**) right-handed circularly and (**b**) linearly polarized incident beam.

In addition, we studied the characteristics of the global SAM and OAM of the output beam by integrals calculations of SAM, OAM in Eqs. (8) and (9) for a series of $\alpha$, and the calculation results are illustrated in Fig. 12.

The global AM could present the controllable conversion between SAM and OAM when we change the angle of the crystals. It’s safe to say that the Fourier transformation would not change the AM of the light field, therefore, the controllable conversion comes from the conical refraction of the light beam in biaxial crystals. And the values of SAM, OAM and AM at $\alpha = 0$ have been proved to be determined by the scaled cylinder, defined by the radio of radius of the cylinder beyond the crystal to the waist of beam^28^. Further increasing the angle between two crystals, the curves showing the change of global AM have different trends for the different SoP of the incident beam.

**Figure 13:**
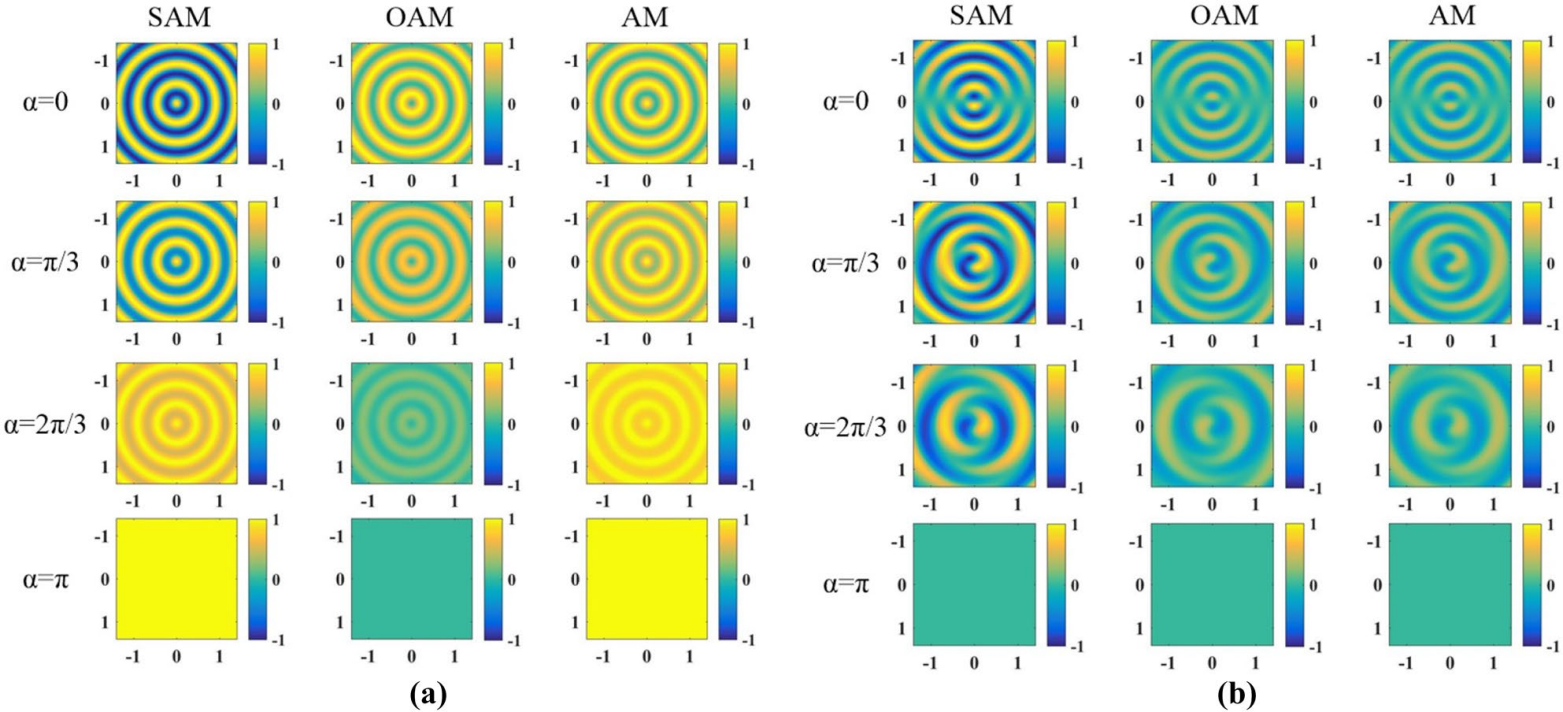
Local SAM, OAM and AM for (**a**) right-handed circularly and (**b**) linearly polarized incident beam.

Figure 13 has shown the calculated local SAM, OAM and AM for right-handed circularly and linearly polarized incident beam. Comparing the Fig. 12 with Fig. 13, we can find that the global AM describes the whole beam by a single value, while the local AM changes in both magnitude and sign over the beam profile. Especially for the linearly polarized incident beam, the global SAM, OAM and AM are all zeros. However, the local SAM, OAM and AM have inhomogeneous spatial distribution, owing to the opposite signs of the local AM in centrosymmetric regions. This non-uniform spin and orbital angular momentum distribution of the light field could generate the optical curl force, as pointed out in Ref.^27^. Our studies demonstrate that changing the angle of the crystals and the SoP of incident beam can control the spatial structure of the polarization, making it feasible to tailor the optical force on a particle.

## Conclusion

In this letter, we have conducted an experiment and measured the spatial distribution of the SoP to verify our previous study. Firstly, the manipulation of Poincaré beams on the different parts of Poincaré sphere is realized in this research. The SoP of incident beam and the angle between the two crystals have influence on the distribution of the SoP of the output beams. Hence, the beams with different SoPs covering the full-Poincaré sphere, part-Poincaré sphere and one point on the sphere are generated in experiment for the different angles between crystals. Besides, the experiment results are in accordance with the theoretical simulation. The errors between the theoretical and experimental results are also explained. For extended research, the spin angular momentum is derived from the distribution of polarization, and meanwhile, the local and global angular momentum are calculated to present the conversion between OAM and SAM. This study realizes the control of the spatial structure of the angular momentum.

The control of the spatial distribution of the polarization will have potential in the polarization-multiplexed free-space optical communication by increasing the channel capacity. This Unitary transformation of light beams may play a role in building the neural networks in the photonic calculation. The non-uniform distribution spin angular momentum of the light field could be associated with the optical curl force of the light field.
